# Achieving Large-Area Hot Embossing of Anti-Icing Functional Microstructures Based on a Multi-Arc Ion-Plating Mold

**DOI:** 10.3390/ma18194643

**Published:** 2025-10-09

**Authors:** Xiaoliang Wang, Han Luo, Hongpeng Jiang, Zhenjia Wang, Ziyang Wang, Haibao Lu, Jun Xu, Debin Shan, Bin Guo, Jie Xu

**Affiliations:** 1 School of Materials Science and Engineering, Harbin Institute of Technology, Harbin 150001, China; 20240206@hit.edu.cn (X.W.); 25b309045@stu.hit.edu.cn (H.L.); jianghp1995@163.com (H.J.); a1437189306@163.com (Z.W.); wzyhit@foxmail.com (Z.W.); shandb@hit.edu.cn (D.S.); guobin@hit.edu.cn (B.G.); 2 National Key Laboratory of Science and Technology on Advanced Composites in Special Environments, Harbin Institute of Technology, Harbin 150080, China; luhb@hit.edu.cn; 3 Shanghai Institute of Spacecraft Equipment, Shanghai 200240, China

**Keywords:** micro-array channels, hot embossing, multi-arc ion coatings, microformability, anti-icing properties

## Abstract

Aluminum alloy surface microstructures possess functional characteristics such as hydrophilicity/hydrophobicity and anti-icing and have important applications in fields such as aerospace and power systems. In order to improve the filling quality of the microstructure and verify the anti-icing property of the microstructure, this work develops a scheme for achieving large-area hot embossing of anti-icing functional microstructures based on a multi-arc ion-plating mold. Compared with conventional steel, the hardness of the PVD-coated steel increases by 44.7%, the friction coefficient decreases by 66.2%, and the wear resistance is significantly enhanced. The PVD-coated punch-assisted embossing could significantly improve filling properties. While the embossing temperature is 300 °C, the PVD-coated punch-assisted embossing can ensure the complete filling of the micro-array channels. In contrast, under-filling defects occur in conventional hot embossing. Then, a large-area micro-channel specimen of 100 cm^2^ was precisely formed without warping, and the average surface roughness Ra was better than 0.8 µm. The maximum freezing fraction of the micro-array channel was reduced by about 53.2% compared with the planar, and the complete freezing time was delayed by 193.3%. The main reason is that the air layer trapped by the hydrophobic structures hinders heat loss at the solid–liquid interface.

## 1. Introduction

Fabricating microstructures on the surface of materials is an effective way to obtain specific functional properties, such as achieving controllable hydrophilicity or hydrophobicity [1], giving materials anti-icing properties [2], improving heat transfer capabilities [3], and giving materials drag reduction properties [4]. As an important engineering material, aluminum alloys have advantages such as low density, high specific strength and specific stiffness, good processability, and high electrical and thermal conductivity. It has significant application prospects in fields such as aerospace, new energy vehicles, power transmission, and shipbuilding. Aviation aircraft operations are accompanied by environmental characteristics such as high humidity, low temperature, and supersaturated water vapor, and it is easy to freeze or even have extreme ice on the surface of the aircraft body, which brings great challenges to operation efficiency and flight safety. Through its unique surface geometric design and chemical characteristics, hydrophobic microstructures can reduce heat transfer and inhibit the initiation of ice crystal nucleation sites [5], delay the icing temperature of supercooled droplets and delay the crystallization process [6], reduce the adhesion of ice layer [7], and then realize surface anti-icing. Wang et al. [8] fabricated a superhydrophobic nanostructured coating on the surface of an aluminum alloy through the chemical method, with a water contact angle exceeding 160°. It was found that corrosion resistance had been improved, and the corrosion inhibition efficiency of aluminum alloy was as high as 99.98%. Jiang et al. [9] developed periodic superhydrophobic micro–nano structures on aluminum alloys using the combination of femtosecond laser processing and multi-step chemical modification, and achieved superior anti-icing and drag reduction properties. The freezing delay time of the micro–nano structured surface was 1.5 times higher than that of an untreated surface, improving fluid flow efficiency and reducing flow resistance by 11.7%. Wang et al. [10] fabricated multi-scale microstructures on 6063 aluminum alloys through micro embossing and hydrothermal treatment, constructing a superhydrophilic surface with a water contact angle of 0°, and achieved dual improvements in boiling heat transfer efficiency and limit, with an increase of over 100% compared with the original surface.

Micro-embossing is a technology that uses plastic deformation to fabricate surface microstructures. It has the advantages of a simple process, high efficiency, low cost, high precision, and superior mechanical properties, which makes it a processing method that can batch fabricate microstructures [11,12]. Fu et al. [13] fabricated the microstructures with a feature size of 100 µm and a maximum aspect ratio of 2 on the surface of stainless steel using micro-embossing technology. He et al. [14] utilized micro-embossing combined with femtosecond laser and subsequent chemical modification to construct superhydrophobic micro–nano structures on the surface of Mg-Li alloys, achieving superior anti-icing and corrosion resistance properties. The freezing time was delayed by 187 s, and the corrosion current density was reduced by 2–3 orders of magnitude. Primeaux et al. [15] used roll forming technology to achieve an efficient and low-cost fabrication of sub-millimeter level micro-array channels and found that the microstructured surface could improve the heat transfer performance between gas and liquid phases. Xu et al. [16] proposed a metal-embossing method based on silicon and tungsten molds, which utilizes ultrafine-grained materials to achieve precise formation of micro-array channels with a minimum width of 5 μm, solving the surface roughening defect in micro-embossing. And combining this with subsequent chemical modification, they realized micro–nano composite structures with superhydrophobicity [17]. In summary, compared with other manufacturing technologies, micro-embossing has significant advantages in process methods and cost control and is a cost-effective surface microstructure manufacturing method.

However, with the continuous integration and enhancement of microstructure functions, the structural characteristics of embossed microstructures have become increasingly complex, the overall area of microstructures has gradually increased, and manufacturing has become more difficult, which could easily lead to mold damage, insufficient filling, warping, and other microforming defects. Primeaux et al. [15] used a cross-rolling method to fabricate micro-array structures, but the secondary rolling caused surface quality defects and channel blockage, leading to the decrease in boiling performance. Significant elastic recovery was observed when the microstructures were fully filled during hot embossing, and while extending the embossing duration or raising the embossing temperature can prevent such recovery, it may lead to severe warping or damage of the embossed parts [18]. Owing to the increased complexity and overall area of microstructures with enhanced performance, it is extremely challenging to achieve defect-free formation using micro-embossing that solely depends on the mold to apply load and temperature. Wang et al. [19] invented an exhaust hot embossing method using a multi-blocked assembled punch for pyramid micro-array structures with three-dimensional complex features, which solved the under-filling and warping defects, but increased the difficulty of mold design and manufacturing. Many scholars are committed to improving microforming performance by using ultrafine-grained materials or applying special energy fields. Xu et al. used ultrafine-grained aluminum with a grain size of 1 μm for micro-embossing, and the fabricated micro-array channels were smoother and more precise compared with those fabricated using coarse-grained materials with grain sizes of several tens of micrometers [16]. Pan et al. [20] introduced an electric field into the embossing process of SUS304 stainless large-area micro-array channels and found that current could reduce stress in the deformation zone of the filling section, as well as improve the filling height and profile quality. Niu et al. [21] established an electric–thermal–mechanical coupled finite element model and studied the electrically assisted micro-embossing process of zirconium alloy surface microstructure. It was found that applying the electric field could increase the depth and quality of microstructures. Yu et al. [22] found that using the softening effect of ultrasonic waves to assist embossing can also improve the microformability. To sum up, using ultrafine-grained materials and special energy field-assisted microforming could significantly improve the plastic flow of materials, reduce embossing loads, and improve the forming quality of microstructures. However, this would lead to a more complex structure of the embossing device, increasing the difficulty of mold design and manufacturing, and the introduction of energy fields would also increase energy consumption.

At present, the formation of ice-resistant surfaces is mostly achieved by subtractive manufacturing technologies such as chemical etching and laser ablation, or by nano-scale structures such as deposition and coating, and most of them are aimed at polymers. There are few studies on microstructures and obtaining ice-resistant surfaces on metal substrates through plastic-forming technology. In the field of microforming, molds are mostly coated with DLC (diamond-like carbon) or uncoated—to avoid microstructure blockage or uneven filling; However, the adhesion between the DLC coating and the substrate is poor, and the internal stress is high [23]. At the same time, the hot forming process of large-area microstructures can easily lead to problems such as warping and surface roughening. Therefore, this study proposes a PVD-coated mold to improve microforming performance and solve the formation problem of large-area functional microstructure composition. Compared with traditional surface strengthening technologies such as electroplating and thermal spraying, PVD coatings have higher hardness and wear resistance, stronger adhesion between the coating and the substrate, and lower surface roughness. Compared with CVD technology [24], PVD coatings have higher impact toughness, and the coating composition can be flexibly adjusted to suit different substrates. Aiming at the core requirements of microstructure forming for high precision, low wear, and long life, PVD can maximize its surface properties without sacrificing punch accuracy, which is difficult to replace with other similar technologies. Among them, a Cr-Zr-N coating can be well combined with die steel substrate. Compared with a Ti-Al-N and Zr/Cr-N coating [25], a Cr-Zr-N coating has better corrosion resistance and stronger impact wear resistance, is more suitable for the hot-pressing formation of aluminum alloy, and can ensure the surface accuracy of workpiece and reduce the die-sticking phenomenon.

In this study, an embossing process assisted by the multi-arc ion-coated punch was proposed to improve the formability of micro-array channels. The hardness and wear resistance of the fabricated multi-arc ion coatings were studied, and the composition and distribution of the coatings on the surfaces of the microstructure punch were analyzed. The microformability of conventional hot embossing and PVD-coated punch-assisted hot embossing was carried out, verifying the feasibility of this process in the trans-scale formation of large-area microstructures, and the anti-icing properties of the embossed micro-array channels was also verified.

## 2. Materials and Methods

### 2.1. Hot Embossing Scheme Based on PVD-Coated Punch

**Figure 1:**
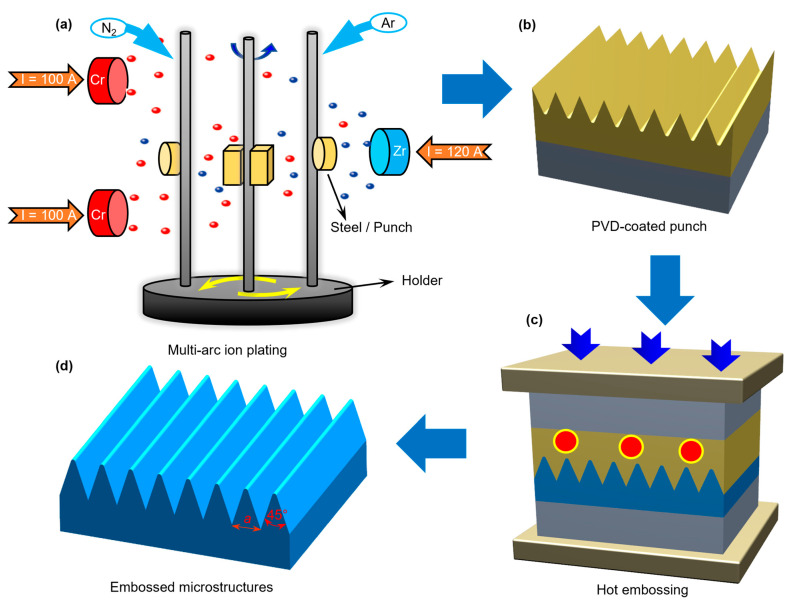
(**a**) Multi-arc ion plating and (**b**) the obtained PVD-coated punch. (**c**) Hot embossing and (**d**) the embossed microstructures.

Figure 1 shows the experimental scheme of this work. Firstly, multi-arc ion plating was used to coat the surface of die steel and microstructure punch with a Cr-Zr-N coating, as shown in Figure 1a. The red and blue dots are respectively chromium ions and zirconium ion released by chromium brick and zirconium brick under the action of electric arc. Then, the PVD-coated microstructure punch was obtained as shown in Figure 1b. Next, hot embossing experimental research on aluminum alloy using PVD-coated punch was conducted. Finally, the embossed microstructures were fabricated, as depicted in Figure 1d, and the outline of the microstructures was triangular micro-array channels, with a side length of 100 µm to 500 µm and a vertex angle of 45°.

**Figure 2:**
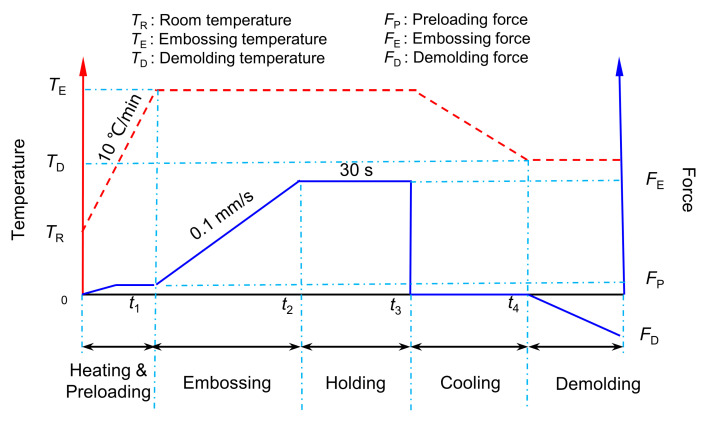
Hot embossing process route.

Specifically, the punch material of the hot embossing punch was made of UNIMAX die steel, which has strong comprehensive properties of thermal strength, toughness, and wear resistance. The coating of the punch was carried out using the multi-arc ion-coating machine (V-Tech CT1000) produced by Dalian V-Tech Nano Technologies Inc (Dalian, China). The process parameters of the multi-arc ion plating were as follows: a vacuum degree of 5 × 10^−3^ Pa, a holding temperature in the furnace of 380 °C, a pulse bias voltage of 120 V, a duty cycle of 80%, a coating time of 80 min, an arc current of the Zr target of 120 A, an arc current of the Cr target of 100 A, an argon gas flow rate of 200 sccm, and a nitrogen gas flow rate of 350~450 sccm. The hot-embossing-forming experiment of the aluminum alloy micro-array channels was carried out using the Systence XT1836-HEB-DX100 (Shanghai Xiteng Electronic Information Technology Co., Ltd., Shanghai, China) precision microforming system with a maximum load of 100 kN. The hot embossing process route is described in Figure 2, and the settings of the embossing temperature and embossing force used in the hot embossing are shown in Table 1. The micro-array channels with side length (a) ranging from 100 µm to 500 µm all adopt the same embossing parameters. According to our previous work experience [19,26], when the embossing temperature was not higher than 200 °C, an embossing force (FE) of 90 kN was applied, whereas when the temperature reached 300 °C, a lower embossing force was used, that is, FE = 40 kN. Other hot-embossing-forming process parameters were as follows: a heating rate of 10 °C/min, an embossing rate of 0.1 mm/s, a holding pressure time of 30 s, and a demolding temperature of 100 °C.

### 2.2. Characterization Method

The HVS-1000A digital micro-hardness tester (Laizhou Huayin Testing Instrument Co., Ltd., YanTai, China) was used to measure the hardness of the die steel before and after multi-arc ion plating. A loading load of 9.807 N was applied each time, a holding time of 10 s was maintained, and the average value was taken after 5 measurements. The wear resistance properties of the die steel before and after plating were tested by the micro-tribometer UMT-2 (CETR Co., El Centro, CA, USA). The wear test was conducted using a Si_3_N_4_ ball head with a diameter of Φ 1.6 mm. A load of 5 N is applied, a rotation speed of 200 r/min is set, the friction time is 600 s, and the friction coefficient is continuously recorded by the micro-tribometer. The PVD-coated punch was polished for 3 h by the cross-section ion-polishing instrument, and the microscopic characteristics of the punch and the composition of the coating were tested by a Zeiss field emission scanning electron microscope. For the micro-array channel specimens after hot embossing formation, the 3D morphological features and surface roughness were measured by a laser confocal microscope (OLS-3000, OLYMPUS, Tokyo, Japan), and the formation quality of the microstructures is observed by a Zeiss field emission scanning electron microscope (Zeiss, Oberkochen, Germany). To evaluate the anti-icing properties of embossed microstructures, a self-made anti-icing test bench was used for testing. The specimen was placed on a semiconductor refrigeration stage at −5 ± 1 °C, and a droplet of 3.0 ± 0.3 μL of water was dropped onto the tested surface. The entire freezing process was recorded using a visual contact angle measuring instrument (JC2000D2, Shanghai Zhongchen Digital Technology Equipment Co., Ltd., Shanghai, China). All data were measured three times and standard deviations were calculated.

## 3. Results and Discussion

### 3.1. Wear Resistance of PVD-Coated UNIMAX Steel

**Figure 3:**
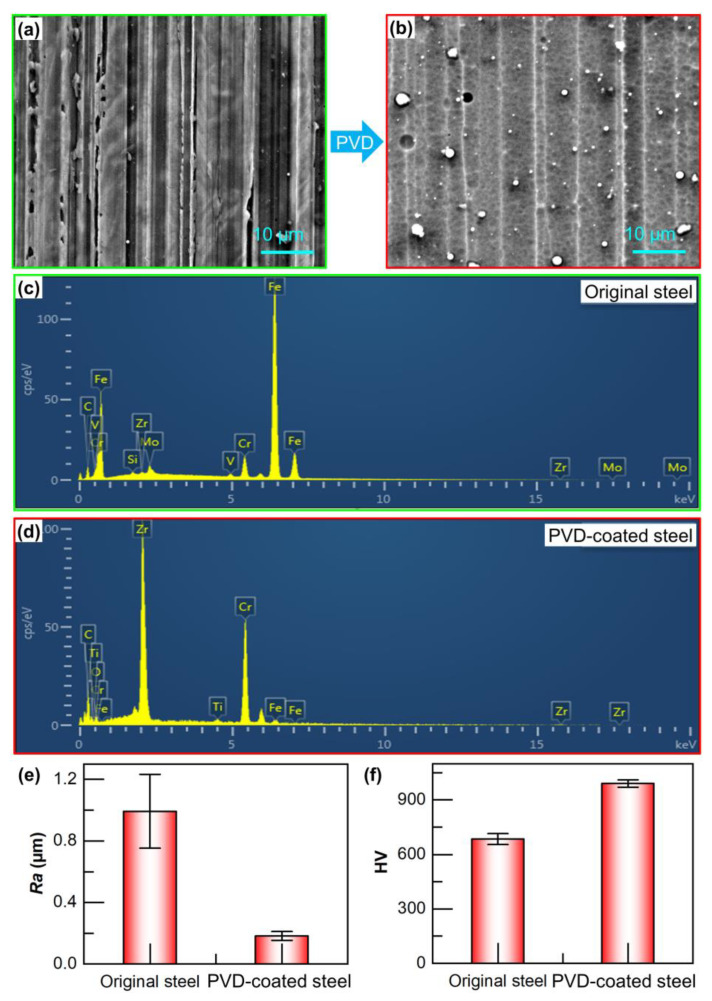
SEM images of (**a**) original and (**b**) PVD-coated steel; EDS spectrum of (**c**) original and (**d**) PVD-coated steel; (**e**) surface roughness and (**f**) hardness before and after multi-arc ion plating.

Due to the fact that the surface of the die punch is arranged with micro-array channels at the sub-millimeter scale and is affected by the assembly structure of the punch itself, it is relatively difficult to test surface hardness and wear resistance. Therefore, the microscopic morphology and hardness and wear resistance of the UNIMAX steel that had undergone multi-arc ion plating in the same batch were tested. The surface quality of the PVD-coated steel obtained after multi-arc ion plating has been significantly improved. It was flatter than the surface of the original steel, as shown in Figure 3a,b. This is mainly because the particles of the Cr and Zr target materials could fill the scratch pits left by the machining, resulting in a smoother surface after multi-arc ion plating [27,28,29]. Figure 3e depicts the measurement results of the surface roughness of the UNIMAX steel before and after multi-arc ion plating. It is found that the surface roughness of the original steel is Ra 0.992 µm, whereas that of the PVD-coated steel is only Ra 0.182 µm. Figure 3c,d show the chemical compositions before and after multi-arc ion plating, respectively, and Table 2 presents the detailed composition. For the original steel, the majority of its composition is Fe. However, after plating, the Fe element on the surface layer of the PVD-coated steel decreases sharply, and the majority consists of Zr and Cr elements, accounting for 55.6 wt% and 41.0 wt%, respectively. Figure 3f shows the measurement results of the hardness. It is found that the hardness of the PVD-coated steel reaches 990.9 HV, which is 44.7% higher than that of the original steel. The original die steel has low inherent hardness and easy undergoes plastic deformation in the hardness test. The multi-arc ion punch is well combined with the substrate through the high-hardness coating, and the coating can resist indentation and suppress indentation expansion when the indenter is in contact. It is the key factor to improve the hardness of die steel by strengthening surface rigidity to make up for the lack of hardness of the soft substrate.

**Figure 4:**
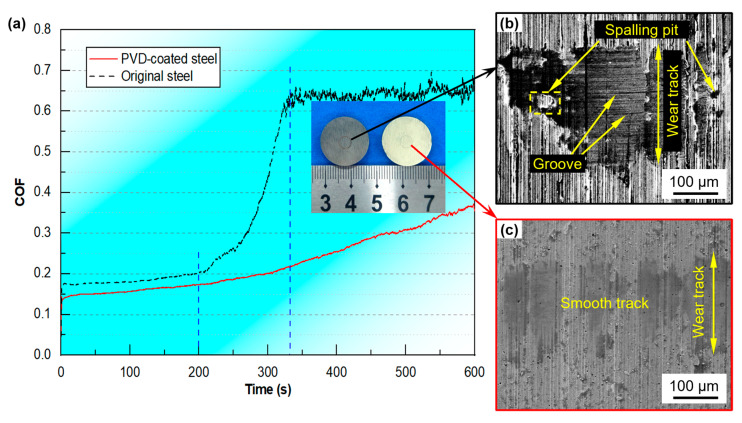
(**a**) Friction coefficient curves before and after multi-arc ion plating. SEM morphologies of the (**b**) original and (**c**) PVD-coated steel after wear.

The higher surface hardness and smoother surface quality of the PVD-coated steel are beneficial for reducing friction during the aluminum alloy embossing process and improving the wear resistance of the punch. The wear resistance of the die steel before and after plating was tested. As shown in Figure 4a, from the wear curve of the coefficient of friction (COF) changing with time, it can be seen that the COF curve of the PVD-coated steel is lower than that of the original steel at any recorded moment, and the trend of the COF curve is smoother. Specifically, for the original steel, within the first 200 s of wear, the COF value slowly increased from approximately 0.18 to 0.20. In the subsequent wear stage, the COF increased very rapidly, reaching approximately 0.65 at 330 s. Subsequently, the friction coefficient remained relatively stable, oscillating between 0.6 and 0.7. In contrast, for the PVD-coated steel, the COF curve was smoother than that of the original steel throughout the entire wear period. Within the first 200 s, the COF slowly increased from approximately 0.16 to 0.18. During the subsequent wear time, it increased from approximately 0.18 to approximately 0.36. At 330 s of wear, the COF of the original steel was close to the peak, whereas the COF of the PVD-coated steel was approximately 0.22, a reduction of 66.2%. Figure 4b,c demonstrate the friction and wear morphologies of the UNIMAX steel before and after multi-arc ion plating, respectively. It is found that the surface of the original steel is significantly damaged after wear, and the friction trace is very rough, and plowing grooves and spalling pits appear on the surface of the steel after wear. However, for the PVD-coated steel, its surface friction trace is smoother than the original steel, and the degree of wear is obviously lighter.

### 3.2. Multi-Arc Ion Plating of Micro-Structured Punches

**Figure 5:**
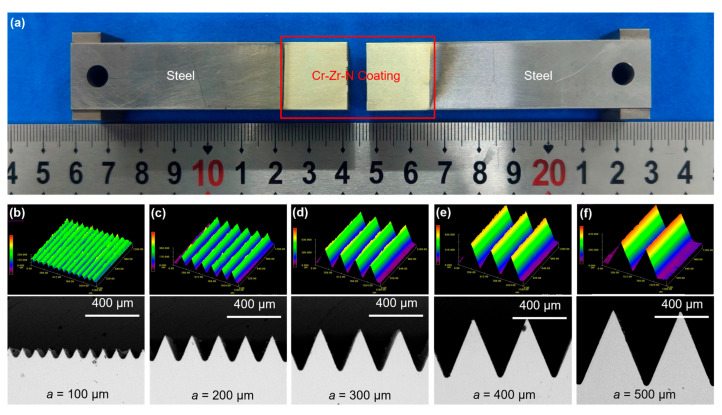
(**a**) Microstructured punches after multi-arc ion plating. Three-dimensional profiles and SEM of PVD-coated punches with micro-channel side lengths of (**b**) 100 µm, (**c**) 200 µm, (**d**) 300 µm, (**e**) 400 µm, and (**f**) 500 µm.

Figure 5 shows the plating results of the microstructured punch. As shown in Figure 5a, the multi-arc ion plating area is the formation area with the micro-array channels and the area that would experience friction with the die cavity during the hot embossing-forming process (about 18 mm away from the part of the micro-array channels). Figure 5b–f, respectively, shows the 3D contours and microscopic morphologies of the micro-channels with side lengths (a) ranging from 100 µm to 500 µm. It is found that the contours of all the micro-channel punches are very regular. For the microstructured punch with the smallest scale (a = 100 µm), the machined arc at the bottom of the micro-array channels is quite obvious, and the surface morphology is rougher than that of other punches, as shown in Figure 5b. On the one hand, due to the very small size of this punch, the proportion of the tool filet is larger compared with the microstructured punches with larger sizes. On the other hand, the micro-array channels of the small-sized punch are more densely arranged and have smaller feature sizes, which need smaller and more precise machining tools, and the cooling and friction conditions during the machining process are worse than those of the machining of the large-sized punch, resulting in lower surface quality [30,31,32]. For microstructured punches with larger scales, the morphology of the micro-channels is fuller. For example, while a = 500 µm, the contour of the micro-array channels is very full and smooth, as shown in Figure 5f.

The values in these subfigures are barely visible, please provide the image of higher resolution in order to make sure it doesn’t affect scientific reading.

**Figure 6:**
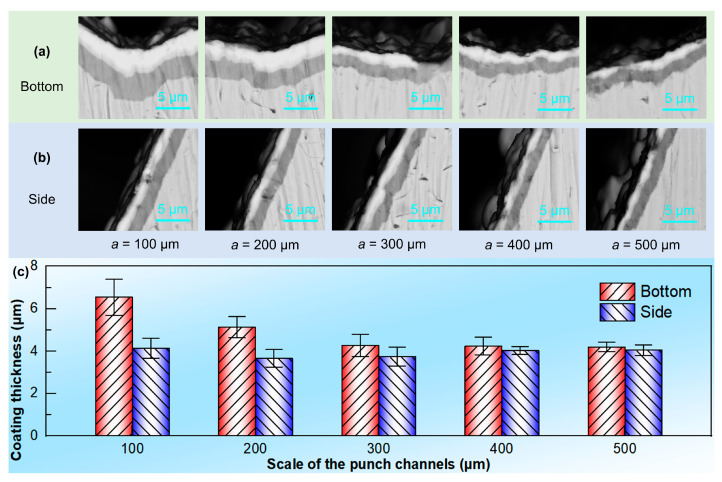
Coating distribution on the (**a**) side and (**b**) bottom surfaces of triangular micro-channel punches. (**c**) Distribution statistics of coating thickness.

Figure 6 shows the distribution of the coating thickness on the bottom and sides of the micro-channels in each punch in Figure 5. The coating thickness of the microstructured punch after multi-arc ion plating is between 4.5 ± 2 µm, and there is a certain relationship between the coating thickness and the scale of the microstructures. As shown in Figure 6a,b, the coating thickness at the bottom gradually becomes thinner as the size of the micro-channels increase, whereas the coating thickness on the side changes little with the increase in the micro-channel size. Moreover, as the size of the micro-channels increases, the coating thickness on the surface of the punch becomes more uniform. Figure 6c describes the variation in the coating thickness on the bottom surface and the side surface of the microstructured punch. Generally speaking, after multi-arc ion plating, the coating thickness on the bottom surface of the micro-channels is greater than that on the side surface, but the difference in coating thickness gradually decreases as the size of the micro-channels increases. Specifically, when a = 100 µm, the coating thickness on the bottom surface of the micro-channels is approximately 6.5 µm, whereas the coating thickness on the side surface is approximately 4.1 µm. When a = 500 µm, the coating thickness on the bottom surface and the side surface of the microchannels is basically the same (the bottom surface of 4.2 µm, and the side surface of 4.0 µm). This is mainly because during the deposition process of the multi-arc ion-plating target material, it tends to accumulate in the pits at the bottom of the micro-array channels, resulting in a thicker coating on the bottom surface than the side surface. The smaller the micro-channel size, the more densely the micro-channels are arranged, and the higher the proportion of the pits at the bottom. The target material is more likely to deposit at the bottom, leading to a thicker coating at the bottom of the smaller sized micro-channels. Nevertheless, on these microstructured punches with the size of several hundred micrometers, the coating thickness is maintained between 4.5 µm ± 2 µm, accounting for a very small proportion, and its influence on the embossing of the micro-array channels could almost be ignored.

**Figure 7:**
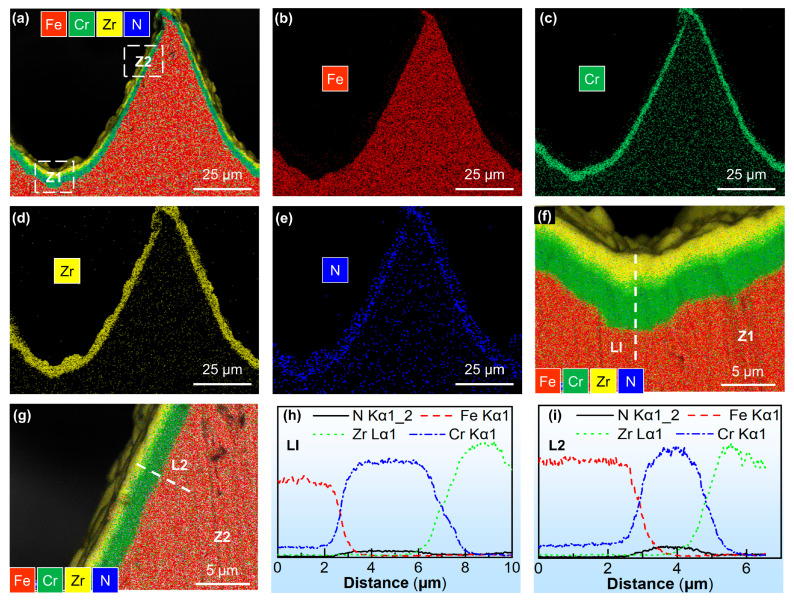
(**a**) EDS results of the cross-section on the microstructured punch. Distribution of (**b**) Fe, (**c**) Cr, (**d**) Zr, and (**e**) N elements. Element distribution of the (**f**) bottom Z1 and (**g**) side Z2 of the micro-channel punch. EDS results on (**h**) bottom line L1 and (**i**) side line L2.

**Figure 8:**
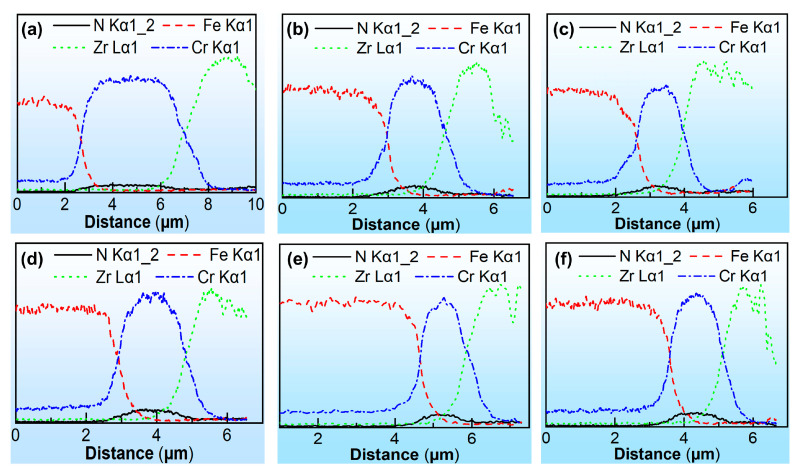
EDS line scanning results on the bottom surface of micro-channel punches with side lengths of (**a**) 100 µm, (**b**) 300 µm, and (**c**) 500 µm. EDS line scanning results on the side surface of micro-channel punches with side lengths of (**d**) 100 µm, (**e**) 300 µm, and (**f**) 500 µm.

The coating composition on the surface of the microstructured punch was further analyzed, as shown in Figure 7 and Figure 8. Taking the microstructured punch with a = 100 µm as an example, it is found that the chemical composition on the surface of the microstructured punch after multi-arc ion plating is mainly that of the Cr-Zr-N layer, as described in Figure 7a. Figure 7b–e, respectively, shows the distribution of Fe, Cr, Zr, and N elements on the surface of the micro-array channels. Each coating element is basically uniformly distributed throughout the micro-channels, and the bottom layer is thicker than the side area. Figure 7f,g, respectively, show the elemental distributions on the bottom surface and the side surface of the micro-channels (as in the selected areas in Figure 7a). Figure 7h,i are, respectively, the compositional analyses of the lines L1 and L2 taken on the bottom surface and the side surface. Since the mass of N is too light and its atomic weight is small, here we mainly consider the Cr and Zr elements. The coating composition, from the inside to the outside, is the Cr layer and the Zr layer in sequence.

For the smaller-scale micro-channels with a = 100 µm, the thickness of the Cr layer at the bottom surface of micro-channels is significantly greater than the Zr layer, whereas there is little difference in the thickness between the Cr layer and the Zr layer on the side surface. When a = 300 µm for the larger-scale micro-channels, whether at the bottom or side surface of the micro-channels, there is little difference in the thickness between the Cr layer and the Zr layer, as shown in Figure 8b,e. When a = 500 µm, the thickness distribution of the Cr layer and the Zr layer is more uniform, as shown in Figure 8e,f. It could be speculated that the coating thickness becomes more uniform with an increase in the size of the micro-array channels, and this phenomenon has a greater relationship with the thickness of the pre-deposited Cr layer. When a = 100 µm, the range of the pre-deposited Cr layer on the bottom surface of the micro-channels is significantly wider than the Zr layer, as shown in Figure 8a. However, as the size of the micro-channels increases, the difference in thickness between the Cr layer and the Zr layer becomes negligible.

### 3.3. Improved Microformability

**Figure 9:**
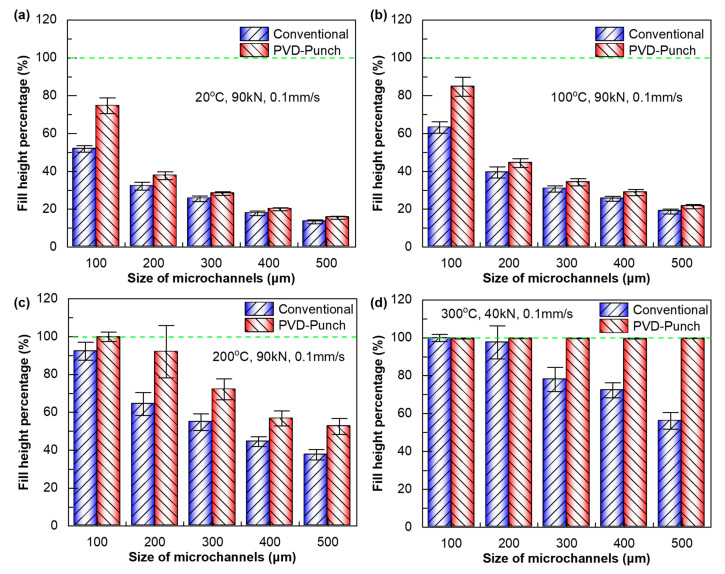
Fill height percentage of embossed micro-array channels with different sizes. (**a**) TE = 20 °C, FE = 90 kN. (**b**) TE = 100 °C, FE = 90 kN. (**c**) TE = 200 °C, FE = 90 kN. (**d**) TE = 300 °C, FE = 40 kN.

To demonstrate the advantages of using multi-arc ion-plating punch to improve the hot embossing formation of micro-array structures, a process comparison was conducted between conventional embossing using uncoated punch and PVD-coated punch-assisted embossing. Figure 9 shows the filling performance of micro-array channels obtained by hot embossing using these two processes. Fill height percentage is used to characterize filling performance, and when the fill height percentage reaches 100%, it indicates that the micro-array channels are fully formed. It is found that under the same embossing parameters, whether in room temperature embossing or hot embossing, the filling performance of the PVD-coated punch-assisted embossing is better than that of conventional embossing. As shown in Figure 9a,b, when TE = 20 °C and 100 °C, and FE = 90 kN, the micro-array channels of all sizes cannot be fully formed, that is, the fill height percentage is less than 100%. The fill height percentage of the PVD-coated punch-assisted embossing is higher than that of conventional embossing. When a = 100 µm, the fill height percentage of the PVD-coated punch-assisted embossing is 21.5% to 22.8% higher than that of conventional embossing. Under the same embossing parameters and at relatively lower temperatures, the fill height percentage of the micro-channels with larger sizes is lower. This is mainly because the deformation amount when micro-channels with larger sizes are fully formed is greater, and they need to undergo more intense plastic deformation and work hardening [33,34]. While the side length of micro-channels increases to 500 µm, the fill height percentage of the PVD-coated punch-assisted embossing is 2.2% to 2.6% higher than conventional embossing.

**Figure 10:**
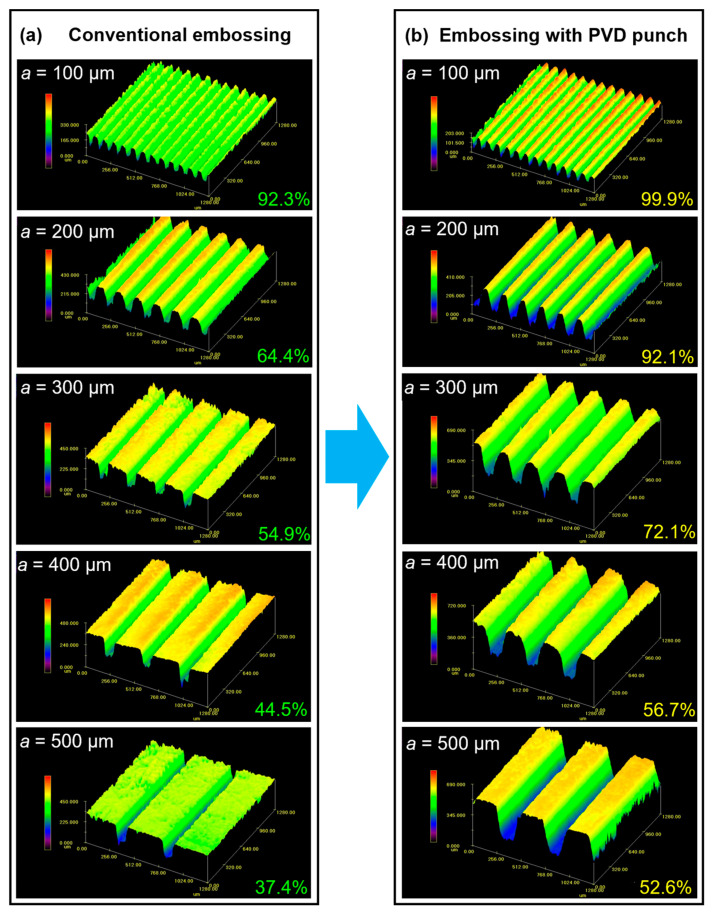
Three-dimensional morphologies of micro-array channels fabricated by (**a**) conventional and (**b**) PVD-coated punch-assisted hot embossing at 200 °C, 90 kN.

As shown in Figure 9c, while the embossing temperature is increased to 200 °C, the filling performance of the aluminum alloy micro-array channels is significantly better than that at TE = 100 °C. Figure 10 shows the 3D contour morphology of the micro-array channels fabricated by hot embossing under these parameters. For the micro-array channels with the smallest size (a = 100 µm), the fill height percentage of both processes exceeds 90%, and the PVD-coated punch-assisted embossing reaches approximately 100%. Similarly, the fill height percentage gradually decreases as the size of the micro-channels increases, but the PVD-coated punch-assisted hot embossing shows higher fill height percentage. In addition, it was found that the PVD-coated punch-assisted hot embossing is more beneficial for the forming of the micro-array channels at high temperatures (TE ≥ 200 °C). For example, while side length ranges from 200 µm to 500 µm at 200 °C, the fill height percentage of the PVD-coated punch-assisted hot embossing is increased by as much as 12.2% to 27.7% compared with that of conventional hot embossing, while at 100 °C, the increase is only 2.6% to 5.0%.

**Figure 11:**
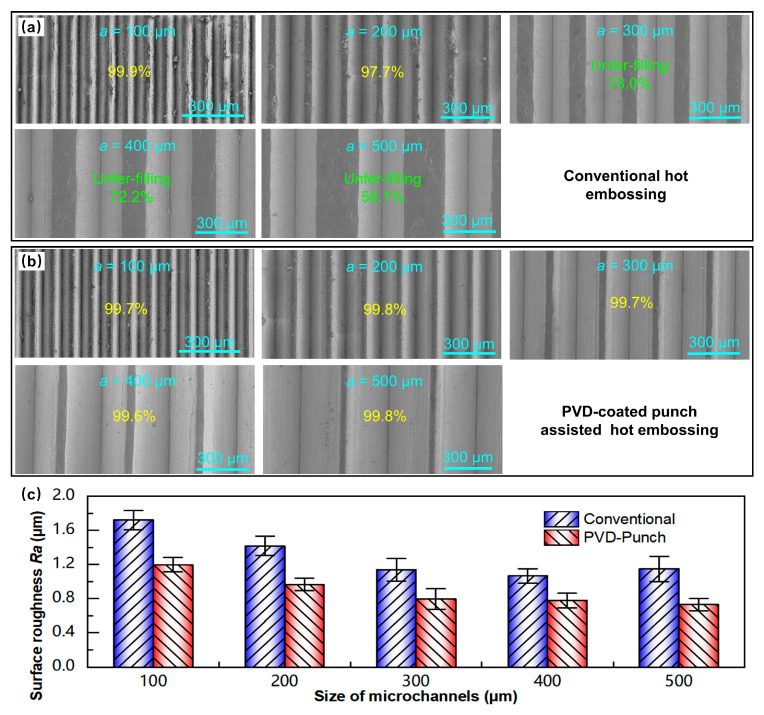
SEM morphologies of micro-array channels formed by (**a**) conventional and (**b**) embossing with PVD-coated punch at 300 °C and 40 kN. (**c**) Average surface roughness comparison.

When the embossing temperature reaches 300 °C, due to the occurrence of dynamic recovery and recrystallization of the aluminum alloy, the material softens severely [35,36]. Although the embossing force is only 40 kN (50 kN lower than that at 200 °C), the filling performance of the aluminum alloy during embossing is significantly improved, and the fill height percentage is also higher, as shown in Figure 9d. Figure 11a,b, respectively, show the SEM morphologies of the micro-array channels fabricated by the two processes under the conditions of 300 °C and 40 kN. For conventional hot embossing, when a = 100 µm and 200 µm, the micro-array channels can be completely filled. As it increases to 300 µm, the micro-channels cannot be fully formed, exhibiting under-filling defects. However, all the micro-array channels of various sizes fabricated by the PVD-coated punch-assisted embossing could achieve complete filling, and the fill height percentage is close to 100%. When a = 500 µm, the fill height percentage of the micro-array channels fabricated by PVD-coated punch-assisted embossing is as high as 99.8%, whereas the conventional hot embossing specimen is only 56.1%. At the same time, the average surface roughness of the micro-array channels formed at 300 °C is compared, and the results are shown in Figure 11c. It is found that the surface of the micro-array channels fabricated by PVD-coated punch-assisted embossing is smoother, and the average surface roughness is lower than conventional embossing. For the micro-array channels fabricated by conventional hot embossing, the average surface roughness Ra ranges from 1.066 to 1.719 µm. Meanwhile, for the specimens fabricated by the PVD-coated punch-assisted embossing, Ra ranges from 0.730 to 1.197 µm, and the roughness is reduced by 27.2% to 36.4%. The wear resistance of the punch with multi-arc ion plating is increased, and its surface is smoother than the punch without coating, as described in Figure 3 and Figure 4; during the embossing and demolding processes, the frictional resistance between the punch and the material is smaller, which is beneficial for the filling of the microstructured punch cavities and the acquisition of a smoother surface quality.

**Figure 12:**
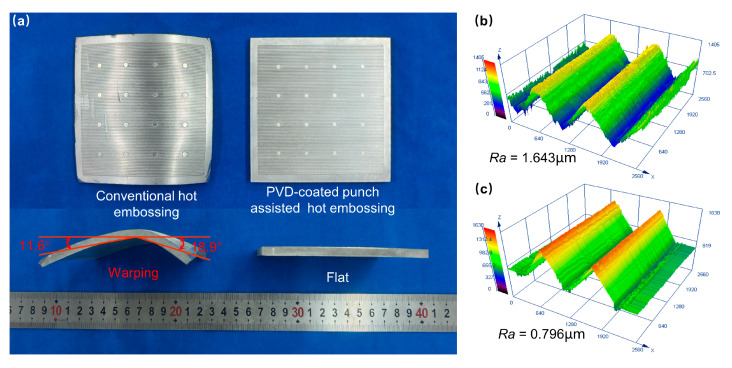
(**a**) Large-area micro-array channels fabricated by conventional and PVD-coated punch-assisted hot embossing. Three-dimensional profiles and average surface roughness of micro-array channels fabricated by (**b**) conventional and (**c**) PVD-coated punch-assisted hot embossing.

To verify the superiority of PVD-coated punch-assisted embossing in large-area trans-scale formation, two processes were used to fabricate large-area micro-array channels with an outer shape size of 100 cm^2^, as shown in Figure 12. It is found that the large-area micro-array channels fabricated by conventional hot embossing suffer severe warping deformation after demolding, whereas the large-area specimen fabricated by PVD-coated punch-assisted embossing is flat, as depicted in Figure 12a. The warping deformation is mainly caused by the excessive friction between the punch and the material during the demolding process [37,38]. The PVD-coated punch after multi-arc ion plating is smoother and has less friction, enabling the warp-free hot embossing formation of large-area micro-array structures. Figure 12b,c show the 3D profiles of the micro-array channels formed by the two processes, respectively. It is found that the large-area micro-channels fabricated by both processes are very full, but the micro-channels fabricated by PVD-coated punch-assisted embossing are smoother, with an average surface roughness Ra of 0.796 µm, which is 51.6% lower than that of conventional hot embossing. Using multi-arc ion plating to coat the surface of the punch with a Cr-Zr-N coating not only improves the hardness and wear resistance of the punch, but also enhances the filling performance and surface quality of the microstructures fabricated by hot embossing. Moreover, this method could be extended to applications in large-area trans-scale formations.

### 3.4. Anti-Icing Properties

**Figure 13:**
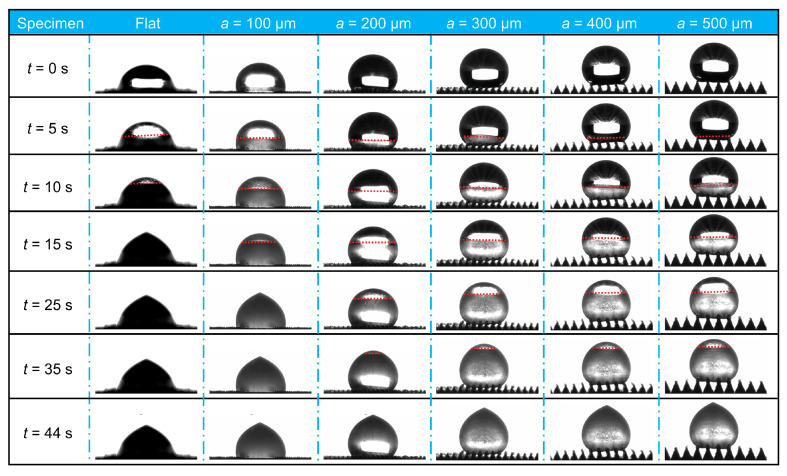
Freezing images of microstructured surfaces at different times: The red dashed line in the figure represents the solid-liquid interface within the droplet. The region above the dashed line is the unfrozen liquid portion, while the region below the dashed line is the frozen solid portion.

**Figure 14:**
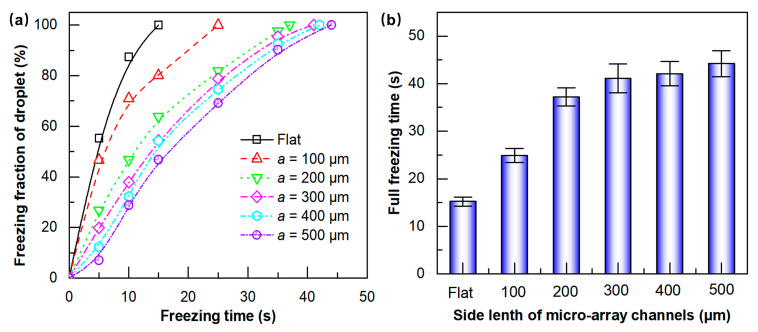
(**a**) The variation in droplet freezing fraction with freezing time; (**b**) influence of micro-array channels on the full freezing time.

Figure 13 shows the changes in the droplet freezing images of various embossed microstructured specimens with freezing times, and the freezing video can be found in the anti-icing properties of microstructured surfaces in the Supplementary Materials. It is found that the anti-icing properties of microstructured surfaces are significantly improved, and all micro-array channel surfaces contributed to delaying icing compared with the flat specimen. Simultaneously, it was observed that the water contact angle on all microstructured surfaces at freezing temperatures was greater than that on flat surfaces, consistent with previous findings at room temperature [26]. Due to air being trapped in the microstructures, the droplet is unable to wet the interior of the microstructures, resulting in an increased contact angle and improved hydrophobicity. Figure 14a depicts the variation in the freezing fractions of each microstructured specimen over time. The droplet freezing fraction of microstructured specimens is lower than that of the flat specimen at any freezing time, and decreases with the increase in the side length of the micro-array channels, indicating an enhancement in anti-icing properties. For the flat specimen, the droplet freezing fraction reached 55.3% within 5 s before freezing, whereas for the micro-array channel surface with a = 100 μm, the droplet freezing fraction was 46.3%. While the side length of micro-channel increased to 500 μm, the freezing fraction was only 7.0%. The freezing fraction increases with time, and the droplet freezing fraction of the flat specimen reaches 100% at 15 s, whereas the surface of the microstructured specimen is still not completely frozen. The freezing fraction of the micro-array channels with the largest scale (a = 500 μm) is only 46.8%, which is 53.2% lower than that of the flat surface. Figure 14b shows the full freezing time of each specimen, and it is found that the full freezing time of all microstructured surfaces is longer than that of the flat and increases with the increase in micro-array channel size. The flat surface completes the entire freezing process at a freezing time of 15 s, whereas the micro-array channel surface with the smallest scale of a = 100 μm completes it at 25 s, with the full freezing time delayed by 66.7% compared with the flat. The maximum scale specimen (a = 500 μm) shows the best anti-icing properties, with the full freezing time delayed by 193.3% compared with the flat and 76.0% delayed compared with the micro-array channels with a side length of 100 μm. The droplet wets the microstructured surface in the Cassie–Baxter state under frozen conditions, and the droplet cannot wet the interior of the micro-array channels, and there is an air layer between them. The presence of the air layer reduces the heat conduction path between the droplet and the surface, increases the heat transfer resistance, and delays heat loss inside the droplet. For microstructures with larger structural scales, the arrangement of microstructures is sparser, the contact area with the droplet is smaller, and the proportion of air layer is higher, resulting in lower solid–liquid or solid–solid heat transfer rates. Therefore, the anti-icing properties of larger-scale micro-array channel specimen are better, the droplet freezing fraction is smaller, and the full freezing time is longer.

**Figure 15:**
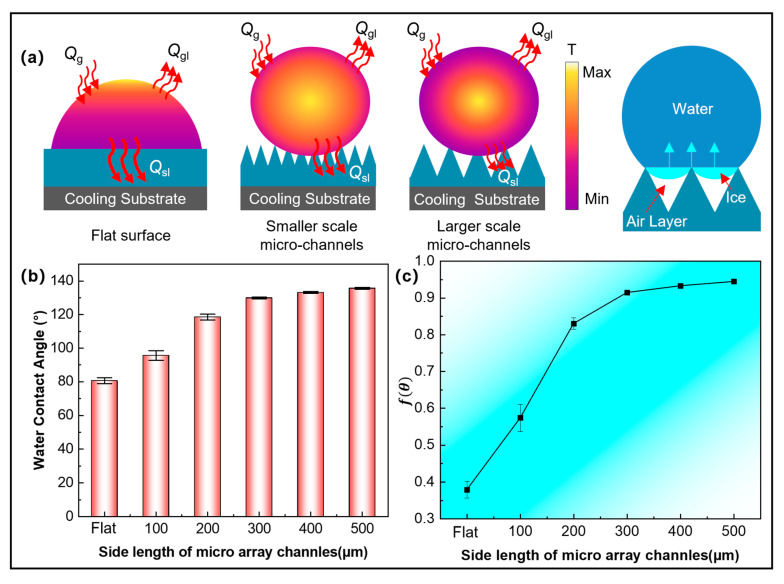
(**a**) Heat transfer models on the micro-array channels; (**b**) water contact angle of micro-array channels; (**c**) geometric correction factor f(θ) of micro-array channels.

The mechanism of enhancing anti-icing properties on micro-array channels is discussed. Under freezing conditions, the droplet resides on the microstructured surface in the Cassie–Baxter state, with the liquid unable to penetrate into the micro-array channels. Instead, a continuous air layer is entrapped between the droplet and the surface. This air layer effectively prevents liquid infiltration into the grooves of the microstructure, thereby increasing thermal resistance at the liquid–solid interface. As a result, the conductive heat transfer pathway between the droplet and the substrate is shortened, reducing the overall heat dissipation from the droplet and delaying its internal cooling, as illustrated in Figure 15a. The process could be quantitatively described by the following equation:

(1) $$∆Q^{\prime }=Q_{g}-(Q_{gl}+Q_{sl})$$

where $∆Q^{\prime }$ represents the heat change rate of the droplet on the micro-array channel per unit time, $Q_{g}$ represents the heat gain absorbed by the droplet from the environment, and $Q_{gl}$ and $Q_{sl}$ correspond to the heat loss through the gas–liquid surface and solid–liquid surface, respectively.

For microstructures with larger structural scales, the microstructure is more sparsely arranged, the contact area with the droplet is smaller, and the proportion of the air layer is higher, resulting in a lower solid–liquid or solid–solid heat transfer rate. This also slows down the speed at which the ice front advances upward from the nucleation point when the droplet freezes. At the same time, the droplet freezing fraction on the larger array microstructure is reduced. When the droplet only contacts the top of the micro-array channel, pinning is formed after freezing, further stabilizing the Cassie–Baxter state. Meanwhile, the Cassie–Baxter state reduces the solid–liquid contact area, thereby reducing the potential heterogeneous nucleation sites during ice crystal nucleation. When the solid–liquid interface reaches the dew point temperature, the lack of nucleation sites prevents the water molecules from being orderly arranged into critical ice nuclei, further maintaining the supercooled state. As the contact angle increases, the effective contact area further decreases, resulting in a decrease in nucleation sites and an increase in supercooling. The relationship between the free energy barrier of the nucleation process and the contact angle (θ) could be calculated as follows [39]:

(2) $$∆G_{hetero}=∆G_{homo}\times f(\theta )$$

where f(θ) is the geometric correction factor, and 0 < f(θ) < 1, considering its simplified form [40]:

(3) f(θ)=(2+cosθ)(1+cosθ)24

Then, the nucleation barrier changes in micro-array channels of different sizes can be calculated, as shown in Figure 15c. As can be seen, f(θ) also increases with increasing micro-array channel size, implying an increase in the nucleation energy barrier. Therefore, droplets in larger micro-array channels must be supercooled more deeply, requiring longer freezing times to overcome the higher energy barrier and initiate ice crystal nucleation. Consequently, samples with larger micro-array channels exhibit better ice resistance, resulting in smaller droplet freezing fractions and longer complete freezing times.

Zhang [41] used laser ablation technology to construct a micro-trench structure superhydrophobic surface (SHS) with a period of 136 μm on the surface of an aluminum alloy, and used 20 μL of water droplets as the research object to test its freezing delay performance in a low-temperature environment of −30 °C. The results show that the freezing time of water droplets on the SHS is 56 s, which is significantly increased by 133.3% compared with the original aluminum plane (24 s). The analysis of its mechanism shows that the freezing delay effect of this surface also originates from the air layer stored in the microstructure gap—this air layer can effectively hinder the heat transfer at the solid–liquid interface, thus delaying the freezing of water droplets, which is completely consistent with the core mechanism of freezing delays caused by thermal insulation of the air layer revealed in this study, and provides cross-research support for the universal mechanism of frost resistance of superhydrophobic surfaces.

## 4. Conclusions

This work proposes a scheme to improve microformability using the punch coated with multi-arc ion plating. The wear resistance of the coating was studied, the coating composition and distribution of the PVD-coated punch were analyzed, the microformability of conventional hot embossing and PVD-coated punch-assisted embossing was compared, and the anti-icing properties of the embossed micro-array channels were tested. The main conclusions are as follows:

(1) After multi-arc ion plating, the average surface roughness of the die steel is reduced from Ra 0.992 µm to Ra 0.182 µm. The hardness is increased by 44.7% compared with the uncoated steel, reaching 990.9 HV, and the coefficient of friction is decreased by 66.2% (at 330 s). The wear morphology shows that there are only slight scratches on the surface of the PVD-coated steel, whereas severe plowing grooves and spalling pits appear on the uncoated steel, indicating a remarkable improvement in wear resistance. Meanwhile, multi-arc ion plating could achieve relatively uniform coatings (with a thickness of 4.5 ± 2 µm) on the punches with micro-array channels at the sub-millimeter scale (100~500 µm), and the coating thickness tends to be more uniform as the size of the micro-array channels increases.
(2) The hot embossing process tests show that PVD-coated punch assisted embossing can significantly improve the filling performance of the micro-channels. At 300 °C, PVD-coated punch-assisted embossing could ensure that micro-channels of all sizes are almost completely filled, whereas conventional hot embossing exhibits under-filling defects. In the verification of large-area trans-scale formation, the 100 cm^2^ large-area micro-channels specimen fabricated by conventional hot embossing undergo severe warping after demolding, whereas the specimen fabricated by PVD-coated punch-assisted embossing is flat, with the average surface roughness Ra of only 0.796 µm, which was 51.6% lower than that of the former.
(3) All embossed micro-array channels could improve surface anti-icing properties, and the anti-icing properties increase with channel size. The anti-icing properties of micro-array channels with the largest side length (500 μm) is the best, with the freezing fraction of 53.2% lower than that of the flat at a freezing time of 15 s, and a full freezing time delay of 193.3%. The main reason is that during the freezing process, the heat of the droplets is mainly lost from the solid–liquid interface. For the microstructure surface, its contact area is smaller, and the heat lost from the solid–liquid interface is less. Meanwhile, the air cushion below the microstructure blocks further heat loss and delays the surface icing time. In summary, this work has quite an important application potential in the surface modification of hot embossing molds and the precise and controllable trans-scale manufacturing of large-area functional micro-array structures.

## Figures and Tables

**Table 1:** Process parameters for hot embossing.

TE (°C)	FE (kN)
20, 100, 200	90
300	40

**Table 2:** Chemical composition of die steel before and after multi-arc ion plating.

Chemical Composition	Zr	Cr	Fe	V, Mo, etc.
Original steel	0.5 wt%	5.6 wt%	90.9 wt%	Bal.
PVD-coated steel	55.6 wt%	41.0 wt%	1.8 wt%	

## Data Availability

The original contributions presented in this study are included in the article/Supplementary Material. Further inquiries can be directed at the corresponding author.

## Supplementary Materials

The following supporting information can be downloaded at https://www.mdpi.com/article/10.3390/ma18194643/s1, Video S1: Anti-icing properties of microstructured surfaces.
